# Sketch of the History of Medicine, from Its Origin to the 19th Century

**Published:** 1835-07-01

**Authors:** 


SKETCH OF THE HISTORY OF MEDICINE, FROM ITS ORIGIN TO THE COMMENCEMENT OF THE NINETEENTH CENTURY. By *J. Bostock*, M.D. 8vo. Sherwood and Co. 1835.

As the history of man is little more than the history of crime—so, that of medicine is little better than a record of follies, and an emblazonment of our shame. As long as this history was prefixed to, or interwoven with medical works on a large scale, and confined to medical readers, there was likely to be advantage derived from the perusal—more perhaps in consequence of the humility taught us, than from any direct knowledge not communicated through the ordinary channels of education. But when these archives of our *conjectural art* are popularized, and diffused through the widening circles of the reading public, we anticipate a rich crop of ridicule to our profession, in addition to that which is daily reaped by most of its members. The following extract from a literary journal, of wide circulation and considerable influence, will amply illustrate our meaning.

" Moliere makes one of his characters declare that 'he knows not a pleasanter farce than that of one man attempting to cure another;' and truly, the perusal of this little volume has gone near to make us of the same opinion. Here is a history of the art from the time of the Assyrians, down almost to the year of grace in which we are writing, from which it clearly appears, that mankind, during that long interval, have lived either wholly without medical advice, or died, dividing their confidence between theorizing pedants and blind empyrics. Systems have changed, methods of cure have flourished and decayed, the most opposite and conflicting remedies have been employed, and men have boasted of their success; but science has continued uninterruptedly its mill-horse circle, covering the same two grains of idea with two bushels of words, and changing only its language from century to century, as if in fear lest men, becoming familiarized with the terms, should detect their emptiness of all meaning, and 'throw physic to the dogs.' "*

And again—

" As a matter of whim, however, and as Jeremy Diddler says, 'just by way of curiosity,' we would ask what the state of medicine is, not in the history of the past, but now in the noonday blaze of the nineteenth century,—divided, as everybody knows it to be, between the homœopathic administrators of quintillionth doses, the antipathic composers of eight-ounce mixtures, and the Mesmerite mystics, not forgetting the charlatanism of the scarifiers *à la* St. John Long, and the unreasoning follow-my-leader-ism of the third and fourth rate prescribers, who form the mass of every-day practitioners?

The plain truth of the matter is, that Physic is merely the younger sister of Theology; and that there is as utter, and as general an incapacity among the patients to give a reason for the faith that is within them, in the one faculty as in the other. The early physicians were professedly, at once, divines, enchanters, and apothecaries; witness the Plutus of Aristophanes, and the history of the impostor Apollonius, by Lucian: even still, traces exist of this union of the natural and the supernatural, in the remnants of judicial astrology among the old-lady herbalist, in the superstition of not cutting the nails on a Friday, &c. *Quo semel est imbuta.*

It is indisputably true, that in latter years, this association has been somewhat severed, and that, since the dissemination of the Baconian philosophy, physiology, the only solid basis of medical reasoning, has made great strides. The physicians of the present age, when they happen to possess common sense, may now argue upon something like established fact. They see and observe with greater accuracy, and draw sounder conclusions from their premises; but still, as far as mere theory is concerned, the *vis dormitiva*, by which Dr. Last explained the efficacy of opium, is the highest flight to which medical philosophy has attained."†

---

* Athenæum, March 28th, 1835. † Ibid.

Such are the reflections excited in the mind of a literary journalist by the perusal of Dr. Bostock's erudite and elaborate "HISTORY OF MEDICINE," and we have no doubt that such will be the effects on most *lay* minds, who have the curiosity to go through the labyrinths of medical theory and practice, as delineated by the talented author of the work before us. We are sorry, therefore, that it has been detached from the "Cyclopædia of Practical Medicine," in which it originally appeared, because it can hardly expect any *additional* diffusion among the profession, in its monographic form, while it may afford pabulum for satire to those extra-professional spirits that delight in ridiculing physic—till they happen to want its assistance.

The history of medicine, itself, pregnant as it is with superstitions, errors, and absurdities, is still a useful portion of study. It enlarges the mind, extends our views, and fixes the great epochs of discovery and improvement more firmly in our memory. To those who are possessed of Le Clerc's History of Medicine, or of Sprengel's History of Medicine, (which brings down the account till the year 1818,) Dr. Bostock's book will be of little use, as containing little or nothing which may not be found in the works alluded to. The materials, however, are neatly abbreviated, and the reflections are happily expressed. As but few, comparatively speaking, possess either Le Clerc or Sprengel, the abstract of medical history drawn up by Dr. Bostock, will prove an acceptable present, and as such we recommend it.

It is curious that Dr. Bostock has stopped short, in his History of Medicine, at the close of the last century, thus leaving 35 years untouched! The short chapter (xiii.) containing "cursory remarks on the state of medicine since the commencement of the present century," contains no attempt at history—and why?—because, "as the historian of medicine approaches nearer to his own times, he finds his path encumbered with almost insurmountable difficulties." We are told by Dr. Bostock that "our actual information does not increase, *in any degree*, in proportion to our experience." Hence, says he, "it follows that the accumulation of materials frequently rather retards than promotes its progress." Now if every historian of medicine were to shirk the quarter or half century that bordered on his own times, how would the history of medicine be carried down from period to period? We strongly suspect that our worthy author broke off, when he found no *other* history of medicine ready cut and dry for him to work upon; and that he did not like the trouble of compiling a history of his own times from original documents. The following passage on the fallacy of experience we shall extract.

"In modern times, and more remarkably in Great Britain, no one thinks of proposing a new mode of practice without supporting it by the results of practical experience. The disease exists, the remedy is prescribed, and the disease is removed; we have no reason to doubt the veracity or the ability of the narrator; his favourable report induces his contemporaries to pursue the same means of cure, the same favourable result is obtained, and it appears impossible for any fact to be supported by more decisive testimony. Yet in the space of a few short years the boasted remedy has lost its virtue, the disease no longer yields to its power, while its place is supplied by some new remedy, which, like its predecessors, runs through the same career of expectation, success, and disappointment.

Let us apply these remarks to the case of fever, the disease which has been styled the touchstone of medical theory, and which may be pronounced to be its opprobrium. At the termination of the last century, while the doctrine of Cullen was generally embraced, typhus fever was called a disease of debility, and was of course to be cured by tonics and stimulants. No sooner was it ascertained to exist, than bark and wine were administered in as large doses as the patient could be induced, or was found able to take. No doubt was entertained of their power over the disease; the only question that caused any doubt in the mind of the practitioner was, whether the patient could bear the quantity that would be necessary for the cure.

To this treatment succeeded that of cold affusion. The high character and literary reputation of the individual who proposed this remedy, its simplicity, and easy application, the candid spirit which was manifested, and the strong testimonials which were adduced by his contemporaries, bore down all opposition, and we flattered ourselves that we had at length subdued the formidable monster. But we were doomed to experience the ordinary process of disappointment; the practice, as usual, was found inefficient or injurious, and it was, after a short time, supplanted by the use of the lancet. But this practice was even more short-lived than either of its predecessors; and thus, in a space of less than forty years, we have gone through three revolutions of opinion with respect to our treatment of a disease of very frequent occurrence, and of the most decisive and urgent symptoms." 229.

Has it never suggested itself to Dr. Bostock's mind that fevers differ very much in their nature, at different periods, and in different constitutions of the atmosphere? We are perfectly certain that we have seen the fevers of one year so different from those which occurred a few years afterwards, as to sanction the differences of treatment alluded to by Dr. Bostock.

In remarking on the utility of medical journals and medical societies, Dr. B. goes rather out of his way to hold up the French Journals as models for us here in England—and he invidiously declares that "the medical periodicals of London are decidedly excelled by those of Edinburgh and Dublin." Now, we believe that WE have never been accused of the attempt to depreciate our contemporaries, either British, continental, or transatlantic, and the remarks which we here make are forced from us by Dr. Bostock's invidious comparison. We tell Dr. Bostock then, that we hold his judgment on the subject, in very low estimation, when he proposes the Parisian journals as models for imitation. Then as to the comparative merits of the medical journals emanating from the three capitals of the united kingdom, how are we to form the estimate? By the extent of the circulation? If Dr. B. knows anything of the matter, he must be aware that, tried by that test, his position is untenable. By republication in foreign lands? In that case his judgment must be impugned. In what way, then, has Dr. Bostock come to his conclusion? Just by prejudice, and by a waspish huff taken against metropolitan medical journals, from some cause or causes best known to himself. Notwithstanding this piece of *ill-judged* judgment, we shall not be so narrow-minded as to depreciate his History of Medicine by comparison with Le Clerc or Sprengel. We leave the comparison to Dr. Bostock's own conscience, for we see ample proof that the author is *intimately* acquainted with the works in question.

